# Factor H–Related Protein 5 Interacts with Pentraxin 3 and the Extracellular Matrix and Modulates Complement Activation

**DOI:** 10.4049/jimmunol.1403121

**Published:** 2015-04-08

**Authors:** Ádám I. Csincsi, Anne Kopp, Miklós Zöldi, Zsófia Bánlaki, Barbara Uzonyi, Mario Hebecker, Joseph J. E. Caesar, Matthew C. Pickering, Kenji Daigo, Takao Hamakubo, Susan M. Lea, Elena Goicoechea de Jorge, Mihály Józsi

**Affiliations:** * Hungarian Academy of Sciences–Eötvös Loránd University “Lendület” Complement Research Group, Department of Immunology, Eötvös Loránd University, 1117 Budapest, Hungary;; † Junior Research Group for Cellular Immunobiology, Leibniz Institute for Natural Product Research and Infection Biology–Hans Knöll Institute, 07745 Jena, Germany;; ‡ Hungarian Academy of Sciences–Eötvös Loránd University Immunology Research Group, Department of Immunology, Eötvös Loránd University, 1117 Budapest, Hungary;; § Sir William Dunn School of Pathology, University of Oxford, Oxford OX1 3RF, United Kingdom;; ¶ Centre for Complement and Inflammation Research, Department of Medicine, Imperial College, London W12 0NN, United Kingdom; and; ‖ Research Center for Advanced Science and Technology, University of Tokyo, Tokyo 153-8904, Japan

## Abstract

The physiological roles of the factor H (FH)-related proteins are controversial and poorly understood. Based on genetic studies, FH-related protein 5 (CFHR5) is implicated in glomerular diseases, such as atypical hemolytic uremic syndrome, dense deposit disease, and CFHR5 nephropathy. CFHR5 was also identified in glomerular immune deposits at the protein level. For CFHR5, weak complement regulatory activity and competition for C3b binding with the plasma complement inhibitor FH have been reported, but its function remains elusive. In this study, we identify pentraxin 3 (PTX3) as a novel ligand of CFHR5. Binding of native CFHR5 to PTX3 was detected in human plasma and the interaction was characterized using recombinant proteins. The binding of PTX3 to CFHR5 is of ∼2-fold higher affinity compared with that of FH. CFHR5 dose-dependently inhibited FH binding to PTX3 and also to the monomeric, denatured form of the short pentraxin C–reactive protein. Binding of PTX3 to CFHR5 resulted in increased C1q binding. Additionally, CFHR5 bound to extracellular matrix in vitro in a dose-dependent manner and competed with FH for binding. Altogether, CFHR5 reduced FH binding and its cofactor activity on pentraxins and the extracellular matrix, while at the same time allowed for enhanced C1q binding. Furthermore, CFHR5 allowed formation of the alternative pathway C3 convertase and supported complement activation. Thus, CFHR5 may locally enhance complement activation via interference with the complement-inhibiting function of FH, by enhancement of C1q binding, and by activating complement, thereby contributing to glomerular disease.

## Introduction

The human complement factor H (FH)–related protein 5 (CFHR5) belongs to the FH protein family (1). This protein family includes two splice variants of the FH gene, FH and FH-like protein 1 (FHL-1), and five CFHR proteins (2). All members of this protein family consist of complement control protein (CCP) domains (also known as sushi domains or short consensus repeats) and show a high degree of sequence similarity to each other and to FH. Whereas FH is a well-characterized inhibitor of the alternative complement pathway, the function of the CFHR proteins is controversial (2, 3). In general, the CFHR proteins lack domains and activity related to the complement regulatory CCP1–4 domains of FH. The C-terminal region is highly conserved within the family: all CFHR proteins possess domains similar to CCPs 19–20 of FH. Some CFHR proteins, for example, CFHR3, contain domains with high similarity to CCP domains 6–7 of FH (2).

**FIGURE 1.**
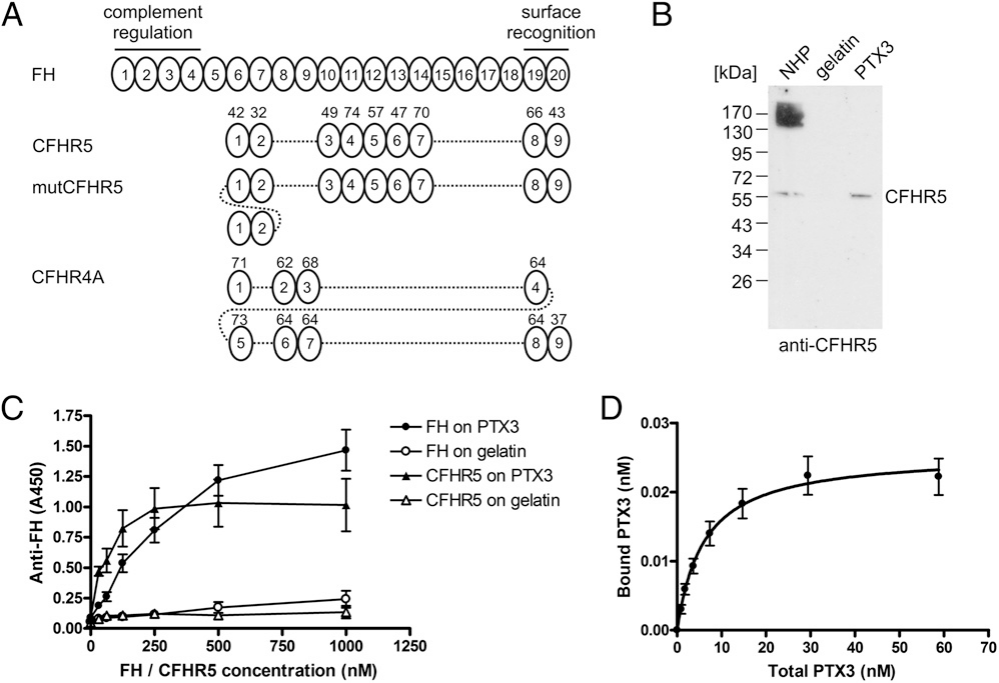
CFHR5 interacts with PTX3. (**A**) Schematic drawing of FH, CFHR5, and CFHR4A. FH is built up of 20 CCP domains, of which CCPs 1–4 mediate complement regulatory activity and CCPs 19–20 mediate surface recognition by FH. The CCP domains of CFHR5, and also CFHR4A, which was used as control protein in several experiments, are shown aligned with the corresponding most related FH domains. The numbers above the domains indicate the percentage of amino acid sequence identity between the homologous domains. The mutant CFHR5 protein detected in CFHR5 nephropathy and containing a duplication of CCPs 1–2 is also shown. CFHR4B consists of CCPs 1 and 6–9 of CFHR4A (not shown). (**B**) Microplate wells were coated with gelatin or PTX3 and then incubated with 25% normal human plasma (NHP). After washing, bound proteins were removed by adding SDS sample buffer and subjected to 10% SDS-PAGE and Western blotting using monoclonal anti-CFHR5. The blot is representative of three experiments. (**C**) Comparison of binding of CFHR5 and FH to PTX3. Immobilized PTX3 and gelatin, used as negative control protein, were incubated with the indicated concentrations of purified FH or recombinant CFHR5. Binding of both proteins was detected by an FH Ab. Data are means ± SEM derived from four experiments. Both FH and CFHR5 bound to PTX3 significantly stronger than to gelatin (*p* < 0.001, two-way ANOVA). (**D**) Addition of increasing amounts of PTX3 results in a saturable binding to immobilized CFHR5. Specific binding was measured using a standard curve of PTX3. Data are means ± SEM from eight experiments.

CFHR5 is a 65-kDa plasma glycoprotein produced in the liver, with a reported serum concentration of ∼3–6 μg/ml (4). CFHR5 consists of nine CCP domains that are related to CCPs 6–7, CCPs 10–14, and CCPs 19–20 of FH (see Fig. 1A). CFHR5 was originally isolated from human glomerular complement deposits (1) with an Ab generated against preparations of a human kidney with membranoproliferative glomerulonephritis (5). Using this Ab, CFHR5 was detected in glomerular immune deposits in several kidney diseases, for example, membranous nephropathy, IgA nephropathy, lupus nephritis, focal glomerular sclerosis, and postinfectious glomerulonephritis (6). Variations in the *CFHR5* gene were also found in patients with atypical hemolytic uremic syndrome and membranoproliferative glomerulonephritis type II/dense deposit disease (7–9). Recently, a subtype of C3 glomerulonephritis was linked to a mutation and internal duplication in the *CFHR5* gene and this disease entity was termed CFHR5 nephropathy (10). The function of the CFHR5 protein is not well understood. It was reported that CFHR5 has cofactor activity for factor I (FI) in the C3b cleavage and CFHR5 accelerates the decay of the fluid-phase C3bBb convertase. However, these activities were only evident at nonphysiological concentrations (4). Analyses of the structural properties of CFHR protein 1 (CFHR1), CFHR2, and CFHR5 revealed that these CFHRs form homo- and heterodimers in serum and can deregulate complement by competing with FH for binding to C3b and surface polyanions (11, 12). Recently, a hybrid CFHR2–CFHR5 protein was shown to cause deregulation of complement (13). CFHR5 was also shown to bind to C-reactive protein (CRP), an acute phase protein belonging to the family of pentraxins (4).

Pentraxins are pattern recognition molecules of the innate immune system and have the capacity to activate complement by binding C1q (14). CRP can interact with several members of the FH protein family, namely FH, FHL-1, CFHR4, and CFHR5 (4, 15, 16), but the interaction of FH with the denatured monomeric CRP versus native pentameric CRP is still a controversial issue (16–20). The long pentraxin, pentraxin 3 (PTX3), is produced locally by neutrophils, macrophages, myeloid dendritic cells, fibroblasts, endothelial cells, and retinal pigment epithelial cells under inflammatory conditions (21, 22). Its plasma level is ∼2 ng/ml, which can increase to ∼1.5 μg/ml during sepsis, inflammation, and infections (23). PTX3 is a 45-kDa glycoprotein and forms stable octamers with disulfide bonds (24). It recruits the complement regulators FH, FHL-1, CFHR1, and C4b binding protein (25–27). Both CRP and PTX3 can initiate complement activation and, by binding complement regulators, govern the reaction to opsonization rather than to the lytic terminal pathway (28). A recent report described PTX3 complexes that also contain CFHR5 in sepsis serum/plasma samples (29).

The aim of this study was to characterize CFHR5–pentraxin interactions, as well as interaction with the extracellular matrix that could be exposed during kidney endothelial injury, and investigate how they influence the regulatory role of FH and activation of complement.

## Materials and Methods

### Proteins, Abs, and sera

Recombinant human FHL-1, CFHR1, CCPs 8–14 of FH (FH8–14), CFHR4A, and CFHR4B were generated using the pBSV-8His baculovirus expression vector (30), expressed in *Spodoptera frugiperda* (Sf9) cells, and purified by nickel-affinity chromatography as described (31, 32). Recombinant human CFHR5, PTX3, anti-CFHR5 mAbs and polyclonal Abs, and biotinylated goat anti-human PTX3 Ab were obtained from R&D Systems (Wiesbaden, Germany). Recombinant mutant CFHR5 with CCPs 1–2 duplicated was produced as described (11). The N- and C-terminal fragments of PTX3 were obtained as previously described (29). The C-terminal fragments of the CFHR proteins were generated as described (11).

Purified human FH, C3, C3b, factor B (FB), factor D, properdin (factor P [FP]), FI, C1q, recombinant human CRP, goat anti-human FH Ab, goat anti-human FB Ab, and goat anti-human C1q Ab were obtained from Merck (Budapest, Hungary). The anti-FH mAb Ab A254 and the anti-FP mAb A235 were from Quidel (Biomedica, Budapest, Hungary). MaxGel, the goat anti-CRP Ab, and the anti–monomeric CRP mAb (mCRP; clone CRP-8) were from Sigma-Aldrich (Budapest, Hungary). The anti-pentameric CRP (pCRP) mAb was purchased from antibodies-online.com (Aachen, Germany). HRP-conjugated goat anti-human C3 was from MP Biomedicals (Solon, OH). HRP-conjugated swine anti-rabbit Igs, rabbit anti-goat Igs, and goat anti-mouse Igs were from Dako (Hamburg, Germany).

Normal human plasma was collected from healthy individuals after informed consent and pooled.

### Microtiter plate binding assays

To analyze binding of native CFHR5 from human plasma to PTX3, 25% normal human plasma, diluted in TBS (10 mM Tris, 140 mM NaCl, 2 mM CaCl_2_, 1 mM MgCl_2_ [pH 7.4]), was added for 1 h at 37°C to wells coated with 5 μg/ml PTX3 or gelatin. After washing, bound proteins were eluted from the wells with SDS sample buffer (60 mM Tris base, 1% SDS, 10% glycerol, bromophenol blue). Eluted proteins were separated on a 10% SDS-PAGE gel and analyzed by Western blot using CFHR5-specific Ab. Binding of CFHR5 and FH to PTX3- and gelatin-coated wells was measured by ELISA using FH Ab and HRP-conjugated anti-goat Ig. TMB Plus substrate (Kem-En-Tec Diagnostics, Taastrup, Denmark) was used to visualize binding, and the absorbance was measured at 450 nm.

To compare PTX3 binding by FH family proteins, Costar microtiter plates (Corning, Corning, NY) were coated with 200 nM each of purified FH, recombinant CFHR1, CFHR4A, and CFHR5 in TBS (25 μl) overnight at 4°C. The wells were washed after each step with TBS containing 0.05% Tween 20. After blocking with 4% dry milk in TBS for 2 h at 37°C, 5 μg/ml PTX3 was added in TBS for 1 h at 37°C. Bound PTX3 was detected with a biotinylated anti-PTX3 Ab followed by HRP-conjugated streptavidin. Calcium and pH dependence of PTX3 binding was analyzed as described previously (27).

To measure PTX3 binding to CFHR protein fragments, wells were coated with 5 μg/ml proteins, followed by blocking with 3% BSA in TBS and incubation with 10 μg/ml PTX3. PTX3 binding was detected as described above.

To compare binding of PTX3 and C3b to the mutant and wild-type CFHR5, the recombinant proteins were immobilized at 10 μg/ml concentration. Binding of 10 μg/ml PTX3 and 10 μg/ml C3b was measured using the corresponding Abs. To compare binding of wild-type and mutant CFHR5 from serum, normal serum and patient serum (11) diluted 1:1 in TBS were applied to wells coated with 10 μg/ml PTX3 and CRP for 30 min at 37°C. After washing, the eluted proteins were analyzed by Western blot using polyclonal CFHR5 Ab as described above.

Binding of C1q to PTX3 was measured by incubating 10 μg/ml C1q added together with increasing amounts of CFHR5 and FH to wells coated with 5 μg/ml PTX3 for 1 h at 22°C. C1q binding was detected using anti-C1q. To measure C1q binding to CFHR5-bound PTX3, microplate wells were coated with 5 μg/ml recombinant CFHR5, with PTX3, and, as control, with gelatin and then incubated with 5 μg/ml PTX3 followed by 25 μg/ml or 50 μg/ml C1q. C1q binding was measured using anti-C1q.

For inhibition assays, wells coated with MaxGel (diluted 1:30 in TBS containing Ca^2+^ and Mg^2+^), PTX3 (30 nM), or CRP (87 nM) were incubated with 50 μg/ml FH in the absence or presence of increasing concentrations of CFHR5 for 1 h at 22°C. FH binding was detected with the mAb A254, which does not recognize CFHR5. To detect competition between CFHR5 and FH in serum, heat-inactivated (56°C, 30 min) human serum was used. Wells were coated with 10 μg/ml PTX3, 10 μg/ml CRP, and MaxGel diluted 1:30 in Dulbecco’s PBS (DPBS). After blocking with 4% BSA in DPBS, the wells were incubated for 30 min at 37°C with 25% heat-inactivated human serum with or without 0.5 μM CFHR5 and CFHR4A. FH binding was detected with mAb A254 and the corresponding secondary Ab.

Interaction of CFHR5 with pCRP was measured in TBS containing Ca^2+^ and Mg^2+^ and with mCRP, which was generated from commercially available CRP as described (33), in DPBS (Lonza, Cologne, Germany). CFHR5 and control proteins were immobilized at 5 μg/ml in microplate wells and, after blocking with 3% BSA in the corresponding buffer, incubated with up to 50 μg/ml pCRP or mCRP. CRP binding was detected with the goat anti-human CRP Ab that recognizes both CRP forms (16). In separate assays, CRP was immobilized in microplate wells at 5 μg/ml in DPBS. Under this condition most bound CRP decays into the mCRP form (16). After blocking, 300 nM CFHR5 (20 μg/ml) and FH (50 μg/ml) were added for 1 h at 22°C and binding was detected using the FH Ab.

### Cofactor assay for C3b inactivation

To assay FH cofactor activity on surfaces, wells coated with MaxGel (diluted 1:30 in TBS containing Ca^2+^ and Mg^2+^), gelatin (10 μg/ml), PTX3 (30 nM), or CRP (87 nM) were preincubated with 20 μg/ml CFHR5 for 1 h at 22°C. After washing, 50 μg/ml FH was added for 1 h at 22°C, followed by thorough washing. Next, 140 nM C3b and 220 nM FI, diluted in TBS, were added in 50 μl to the wells and incubated for 1 h at 37°C. The reactions were stopped by adding reducing SDS sample buffer. Samples were then loaded onto 10% SDS-PAGE gels, separated by electrophoresis, and subjected to Western blot. C3 fragments were detected using HRP-conjugated goat anti-human C3 and an ECL detection kit (Merck).

### Extracellular matrix assays

To study the binding of CFHR5, PTX3, and FH to human extracellular matrix (ECM), MaxGel diluted 1:50 in TBS was immobilized on microtiter plate wells overnight at 4°C and used for subsequent binding assays (as described above). Endothelial cell–derived ECM was prepared as described (25, 27) by culturing HUVEC (American Type Culture Collection; LGC Promochem, Wesel, Germany) on gelatin-coated 96-well tissue culture plates (0.2% gelatin) in DMEM (Lonza) supplemented with 10% FCS, 1% l-glutamine, and 50 μg/ml gentamicin sulfate in a cell incubator with humidified atmosphere containing 5% CO_2_ for 7 d at 37°C. Cells were washed and detached from the plate by incubation in DPBS containing 10 mM EDTA at 37°C. Removal of the cells was monitored by microscopy. The cell-free ECM was washed with TBS and used immediately for binding assays as described above. The production of ECM by endothelial cells was confirmed by detecting ECM components after cell detachment, using Abs against laminin, collagen type IV, and von Willebrand factor (Sigma-Aldrich).

### C3 convertase assays

Formation of the C3bBb alternative pathway C3 convertase on surface-bound CFHR5 and detection of the C3 convertase components were performed as previously described (34). The convertase activity was measured by adding 10 μg/ml purified C3 for 1 h at 37°C and quantifying the generated C3a by a C3a ELISA kit (Quidel).

### Complement activation assays

To measure complement activation due to competition between CFHR5 and FH for ligand binding, Nunc microplate wells were coated with 10 μg/ml PTX3, 10 μg/ml CRP, and MaxGel diluted 1:30 in DPBS. After blocking with 4% BSA in DPBS, 12.5% normal human serum (for PTX3 and CRP) and 25% normal human serum (for ECM) were added for 30 min at 37°C, with or without 20 μg/ml CFHR5 or CFHR4A. Complement activation was detected by measuring deposition of C3 fragments using HRP-conjugated goat anti-human C3.

In other experiments, Nunc microtiter plate wells were coated with 5 μg/ml CFHR5, CFHR4A, FH, and human serum albumin (HSA) and, after blocking with 3% BSA in DPBS, incubated with 10% normal human serum with or without 5 mM Mg^2+^-EGTA or 5 mM EDTA for 30 min at 37°C. Deposition of C3b, FB, and FP was detected using the corresponding primary and secondary Abs.

### Statistical analysis

Statistical analysis was performed using GraphPad Prism version 4.00 for Windows (GraphPad Software, San Diego, CA). A *p* value <0.05 was considered statistically significant.

## Results

### CFHR5 binds to PTX3

Previously, we showed that serum FH and CFHR1 bind to PTX3 (27). To investigate binding of CFHR5, we incubated human plasma in PTX3-coated wells and analyzed the bound proteins after elution and SDS-PAGE by Western blotting using CFHR5-specific Ab. The native CFHR5 protein was detected by this approach in the PTX3-coated wells but not in gelatin-coated control wells (Fig. 1B). Direct interaction of CFHR5 with PTX3 was analyzed by ELISA. Recombinant CFHR5 showed dose-dependent binding to PTX3, and the binding of CFHR5 to PTX3 reached saturation at a lower concentration compared with the binding of FH (Fig. 1C). To further analyze this, serial dilutions of PTX3 were applied to CFHR5, immobilized in microplate wells, and the amount of bound PTX3 was calculated from a standard curve (Fig. 1D). Dose-dependent binding was observed in the 1–10 nM range; however, avidity effects complicate affinity determinations because of the octameric nature of PTX3 and CFHR5 being dimeric, resulting potentially in multivalent binding.

### Characterization of the CFHR5–PTX3 interaction

**FIGURE 2.**
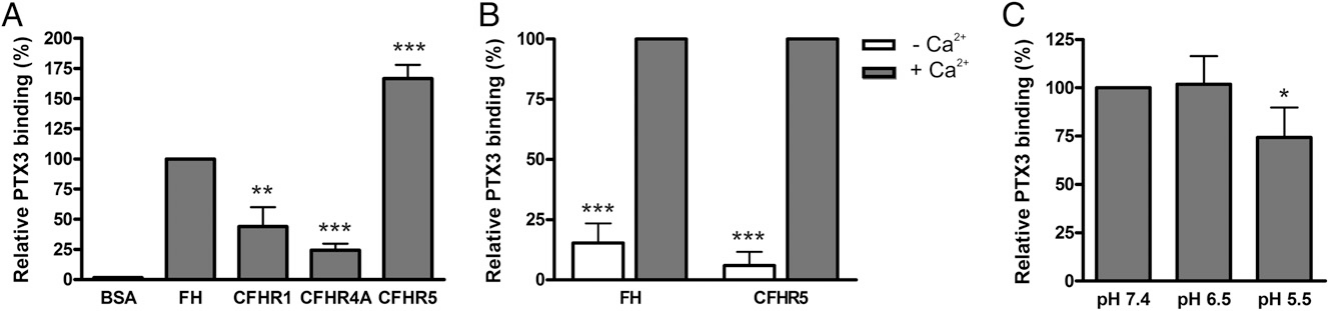
Comparison of the binding of PTX3 with CFHR proteins, and dependence of the PTX3–CFHR5 interaction on calcium concentration and pH. (**A**) Binding of PTX3 to recombinant CFHR5 was compared with that of CFHR1, CFHR4A, and FH by ELISA. The FH family proteins and BSA, used as negative control, were immobilized in equimolar concentrations (200 nM) in microplate wells, and then 5 μg/ml recombinant PTX3 was added for 1 h at 37°C. PTX3 binding was determined using a polyclonal anti-PTX3 Ab. The values were normalized for FH binding (100%) and show means ± SD derived from three independent experiments. **p < 0.01, ***p < 0.001, one-way ANOVA. (**B**) The binding of PTX3 to immobilized FH and CFHR5 was compared in DPBS (pH 7.4) with (filled bars) and without (open bars) 1 mM Ca^2+^. The data are normalized to binding in the presence of Ca^2+^ (100%) and represent means ± SD from four experiments. ***p < 0.001, one-way ANOVA. (**C**) The binding of PTX3 to CFHR5 was compared in TBS with pH 7.4, 6.5, and 5.5. The data shown are normalized to values obtained with TBS pH 7.4 (100%) and represent means ± SD from five experiments. *p < 0.05, one-way ANOVA.

To compare the binding of PTX3 to CFHR proteins, equimolar amounts of purified FH and recombinant CFHR1, CFHR4A, and CFHR5 were immobilized in microplate wells and PTX3 was added in the fluid phase. CFHR1 showed less PTX3 binding capacity compared with FH, in agreement with our previous results (27), whereas CFHR5 showed the strongest binding among the studied proteins (Fig. 2A). PTX3 binding to CFHR4A was rather weak, similar to that reported previously for CFHR4B (26).

Because the presence of Ca^2+^ is required for several interactions of pentraxins, the role of Ca^2+^ in the binding of PTX3 to CFHR5 was studied. Similar to FH, CFHR5 showed strongly reduced PTX3 binding when Ca^2+^ was absent from the buffer (Fig. 2B).

The binding of PTX3 to FH and FHL-1 was shown to be increased at lower pH (27). In contrast, we found that the binding of CFHR5 was slightly but significantly reduced at pH 5.5 compared with the physiological pH (Fig. 2C).

### Analysis of binding sites involved in the CFHR5–PTX3 interaction

**FIGURE 3.**
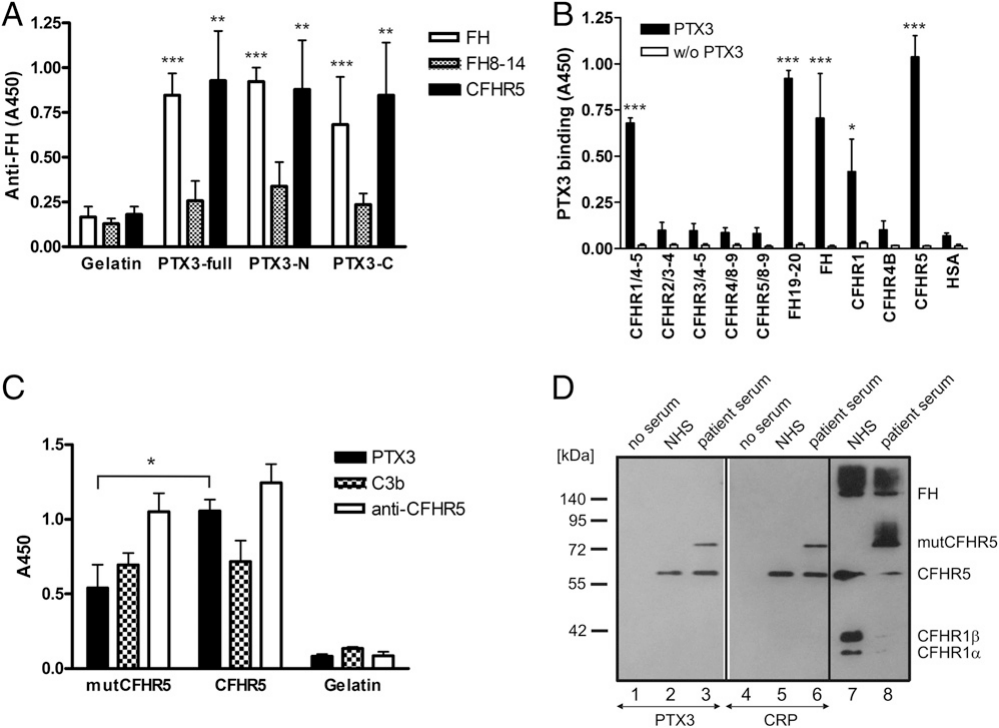
Characterization of the PTX3–CFHR5 interaction. (**A**) Gelatin, full-length PTX3, and the N- and C-terminal PTX3 fragments were immobilized in microplate wells (5 μg/ml). Binding of FH (40 μg/ml), FH8-14 (10 μg/ml), and CFHR5 (10 μg/ml) was measured using polyclonal anti-FH Ab. Data are mean absorbance values ± SD derived from four independent experiments. **p < 0.01, ***p < 0.001, one-way ANOVA. (**B**) Binding of PTX3 to the C-terminal CFHR domains that are the homologs of the PTX3-binding CCP19–20 of FH was measured by ELISA. CCP4–5 of CFHR1, CCP3–4 of CFHR2, CCP4–5 of CFHR3, CCP8–9 of CFHR4A, CCP8–9 of CFHR5, and CCP19–20 of FH, as well as the full-length FH, CFHR1, CFHR4B, CFHR5, and HSA proteins as controls, were immobilized at 5 μg/ml concentration in microplate wells. Recombinant PTX3 at 10 μg/ml was added and its binding was detected as described for Fig. 2. Data are mean absorbance values ± SD derived from three independent experiments. *p < 0.05, ***p < 0.001, one-way ANOVA. (**C**) Binding of PTX3 to the CFHR5 mutant with duplicated CCP1–2 (mutant FH–related protein 5 [mutCFHR5]) was measured by immobilizing mutCFHR5, and as controls, CFHR5 and gelatin at 10 μg/ml in microplate wells. Binding of 10 μg/ml PTX3, 10 μg/ml C3b, and a CFHR5-specific polyclonal Ab was measured by ELISA as described in *Materials and Methods*. The data are means ± SD derived from four independent experiments. *p < 0.05, one-way ANOVA. (**D**) Wells coated with PTX3 (*lanes 1–3*) and CRP (*lanes 4–6*) were incubated with 50% normal human serum (NHS) (*lanes 2* and *5*) and a patient serum containing both wild-type CFHR5 and the mutCFHR5 (*lanes 3* and *6*). The bound proteins were analyzed after elution and 10% SDS-PAGE by Western blotting using polyclonal CFHR5 Ab. Reactivity of the polyclonal Ab with NHS (1 μl, *lane 7*) and patient serum (1 μl, *lane 8*) is shown as a control. The blot is representative of two experiments.

To determine which domain of PTX3 mediates the binding to CFHR5, recombinant N- and C-terminal PTX3 fragments were immobilized on microtiter plates and incubated with recombinant CFHR5. CFHR5 bound to both the N- and C-terminal parts of PTX3 (Fig. 3A). FH also bound to both PTX3 fragments, whereas the control recombinant CCP8–14 domains of FH did not bind to any PTX3 fragment, in agreement with previous results (26).

Because the CCP19–20 domains of FH were shown to be involved in binding PTX3 (26, 27), we investigated the capacity of the homologous CCP8–9 domains of CFHR5, and also the homologous domains of the other CFHR proteins, to bind PTX3. PTX3 did not bind to the CFHR5 CCP8–9 fragment, indicating a binding site outside these domains (Fig. 3B). PTX3 bound to CCP19–20 of FH and CCP4–5 of CFHR1, as expected from previous studies (26, 27), but it did not bind to CCP3–4 of CFHR2, CCP4–5 of CFHR3, and CCP8–9 of CFHR4A (Fig. 3B).

A disease-associated CFHR5 protein contains CCPs 1–2 in two copies (Fig. 1A). To analyze the role of these domains and the capacity of this disease-associated CFHR5 mutant (mutant FH–related protein 5 [mutCFHR5]) in PTX3 binding, the binding of PTX3 to immobilized recombinant mutCFHR5 protein compared with wild-type CFHR5 was measured. The mutCFHR5 protein bound less PTX3 than did the wild-type protein, whereas the binding of fluid-phase C3b was comparable to both proteins in the assay (Fig. 3C).

The interaction was confirmed using serum as a source of CFHR5 (Fig. 3D). Normal human serum and a patient serum containing both wild-type CFHR5 and the mutCFHR5 were incubated in wells coated with PTX3 and the related short pentraxin CRP. The bound proteins were analyzed after elution and SDS-PAGE by Western blotting using polyclonal CFHR5 Ab. Whereas the amount of the mutCFHR5 was clearly higher in the patient serum compared with the normal CFHR5 (Fig. 3D, *lane 8*), a weaker mutCFHR5 band was observed among both the PTX3- and CRP-bound proteins, confirming the results obtained with recombinant mutCFHR5.

### Effect of the ligands C3b and C1q on the CFHR5–PTX3 interaction

The main known CFHR5 ligand is the complement fragment C3b. Therefore, we studied whether C3b influences the PTX3 binding capacity of CFHR5. To this end, CFHR5 immobilized in microplate wells was preincubated with increasing concentrations of C3b. Then PTX3 was added and PTX3 binding was measured. C3b showed dose-dependent binding to CFHR5 and it did not influence the binding of PTX3 (5 μg/ml) to CFHR5 at concentrations up to 50 μg/ml (data not shown).

**FIGURE 4.**
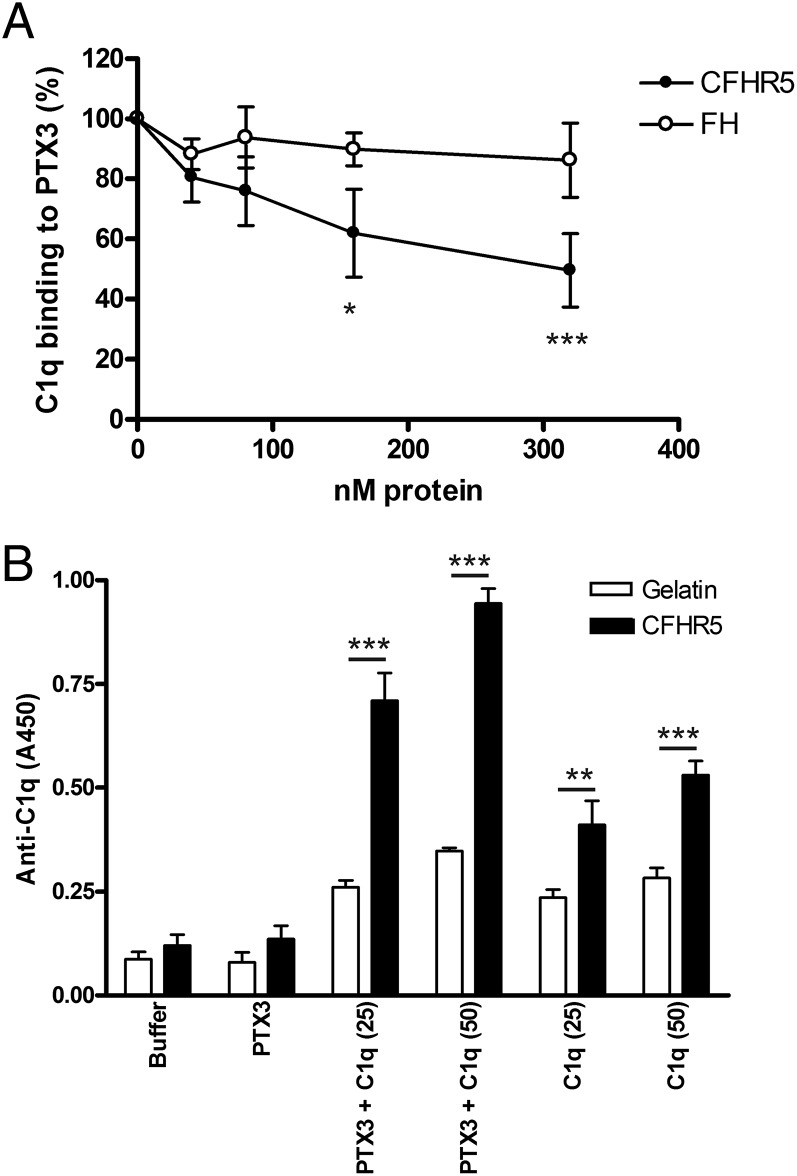
C1q binding to PTX3 in the presence of CFHR5. (**A**) Binding of C1q to PTX3 was determined in the presence of increasing concentrations of CFHR5 and FH in ELISA. C1q binding was detected using C1q Ab. The normalized values are means ± SD derived from four independent experiments. *p < 0.05, ***p < 0.001, two-way ANOVA. (**B**) C1q binds to CFHR5-bound PTX3. Microplate wells were coated with 5 μg/ml recombinant CFHR5 and, as control, with gelatin, and then sequentially incubated with 5 μg/ml PTX3 and 25 μg/ml or 50 μg/ml C1q, as indicated. C1q binding was measured using C1q Ab. The data are means ± SD derived from four independent experiments. **p < 0.01, ***p < 0.001, one-way ANOVA.

C1q binding to PTX3 is thought to initiate classical complement pathway activation (35). We investigated whether CFHR5 can interfere with the binding of C1q to PTX3. To this end, wells coated with PTX3 were preincubated with increasing amounts of CFHR5 and C1q binding was measured. CFHR5 could partly inhibit C1q binding to PTX3 (up to ∼50% under the tested experimental conditions), whereas FH had no inhibitory effect on C1q binding to PTX3 (Fig. 4A). In a reverse setting, we analyzed whether CFHR5-bound PTX3 can interact with C1q. C1q showed binding to CFHR5 that was strongly enhanced by PTX3 bound to CFHR5 (Fig. 4B). Thus, C1q can bind to CFHR5-bound PTX3.

### CFHR5 competes with FH for binding to PTX3

**FIGURE 5.**
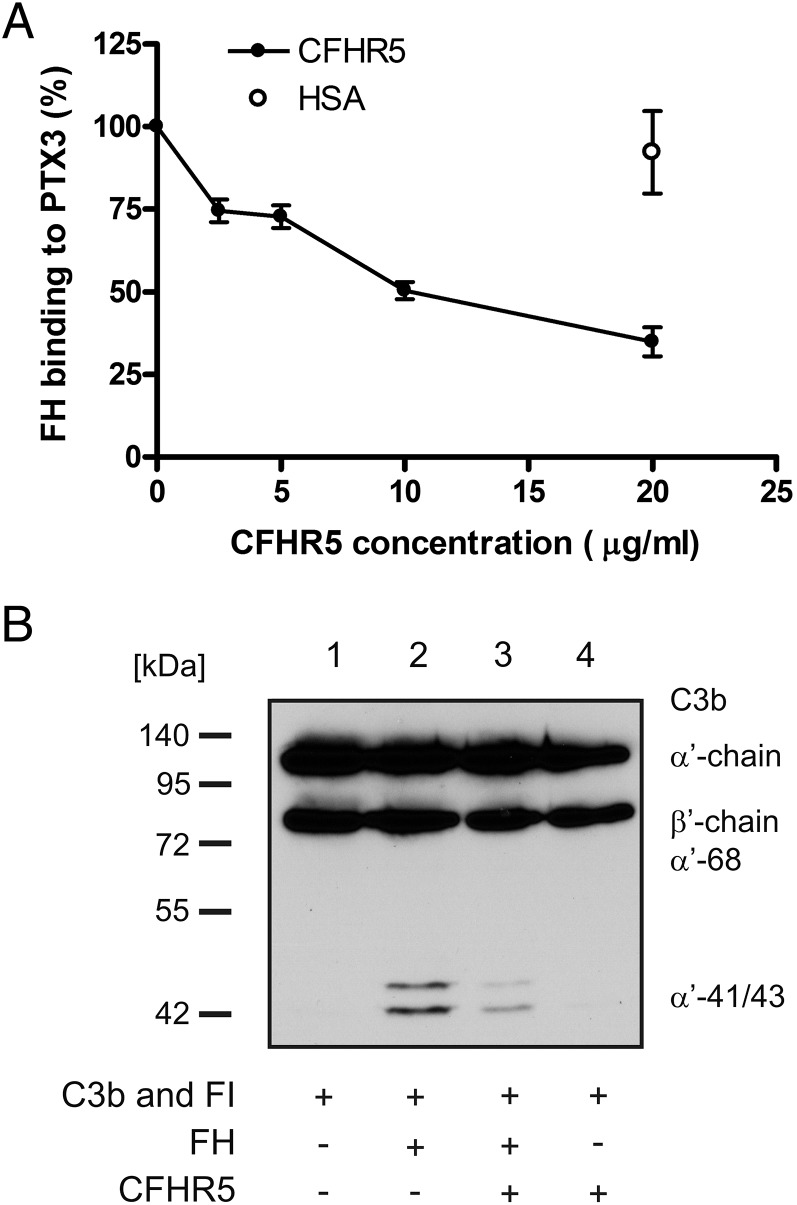
CFHR5 competes with FH binding and activity on PTX3. (**A**) The binding of 50 μg/ml (∼300 nM) FH to PTX3 was measured in the presence of increasing concentrations of CFHR5 (up to 20 μg/ml, corresponding to ∼300 nM CFHR5) using an FH-specific mAb for detection. HSA was used as control protein. The normalized values are means ± SD derived from four independent experiments. The binding of FH was significantly different in the presence of 20 μg/ml CFHR5 than in the presence of 20 μg/ml HSA (*p* = 0.0005, unpaired *t* test). (**B**) The cofactor activity of FH bound to PTX3 for the cleavage of C3b in the presence and absence of CFHR5 was measured by Western blot as described in *Materials and Methods*. Microplate wells were coated with 10 μg/ml PTX3 and incubated with 50 μg/ml FH with or without 20 μg/ml CFHR5, as indicated below the blot, and then C3b and FI were added to each well. The molecular mass marker is indicated on the *left*, and the C3b chains and the C3b α′-chain cleavage fragments are indicated on the *right*. The blot was developed using HRP-conjugated C3-specific Ab that recognizes C3b and its fragments but not C3d. The blot is representative of three independent experiments.

The binding of FH to PTX3 is thought to downregulate PTX3-induced complement activation (26, 27). Therefore, we assessed the ability of CFHR5 to interfere with the binding of the complement inhibitor FH to PTX3. CFHR5 strongly and dose-dependently inhibited FH binding to PTX3, whereas the control protein HSA had no effect on this interaction (Fig. 5A). Accordingly, CFHR5 inhibited the cofactor activity of PTX3-bound FH by strongly reducing FH binding to PTX3. When bound to PTX3, FH acted as a cofactor for the FI-mediated cleavage and inactivation of C3b (Fig. 5B, *lane 2*), which was strongly reduced by CFHR5 added in 1:1 molar ratio to FH (300 nM each) (Fig. 5B, *lane 3*), whereas CFHR5 itself showed no cofactor activity (Fig. 5B, *lane 4*).

### CFHR5 binds to denatured, monomeric CRP and inhibits FH binding and cofactor activity

**FIGURE 6.**
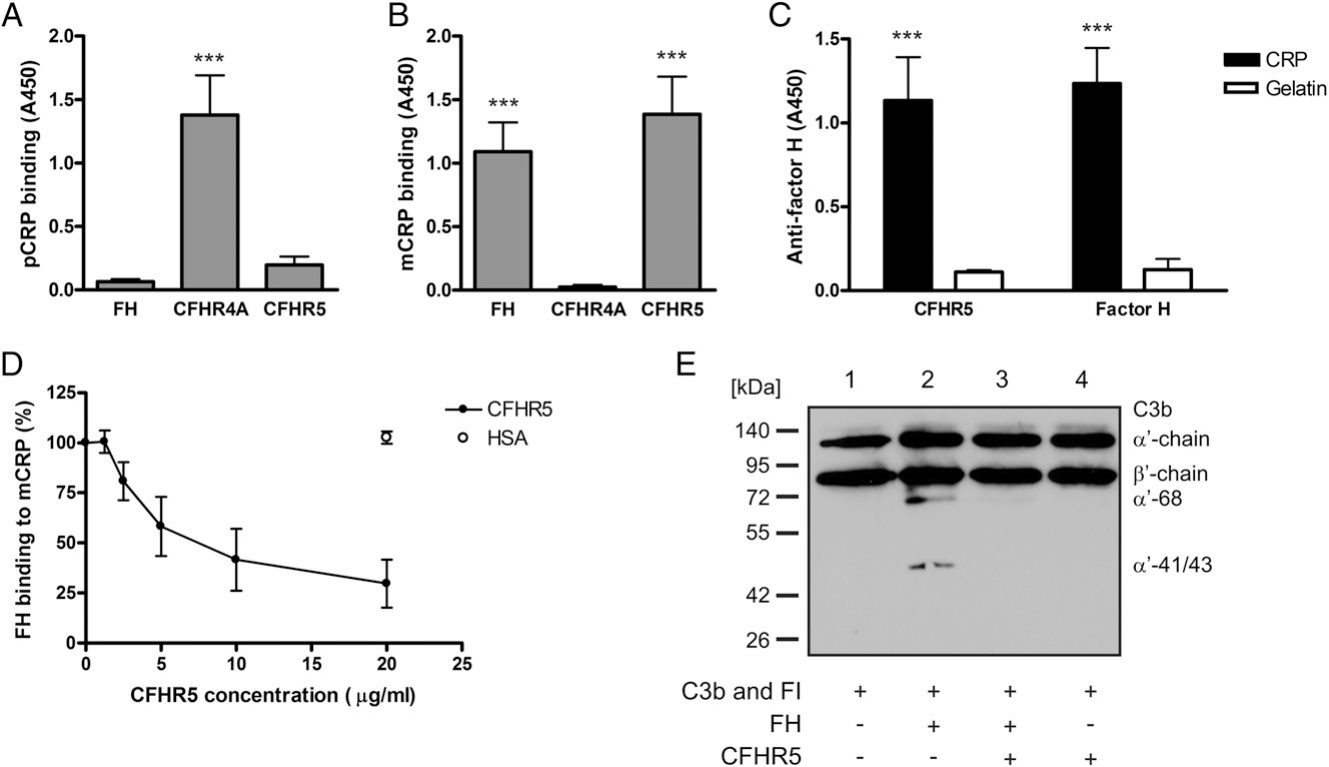
Interaction of CFHR5 with CRP. (**A**) FH, CFHR4A, and CFHR5 were immobilized in microplate wells at 5 μg/ml. After blocking, binding of 50 μg/ml pCRP was measured in TBS containing 2 mM CaCl_2_ and 1 mM MgCl_2_ using a polyclonal CRP-specific Ab. Data are mean absorbance values ± SD from three experiments. ***p < 0.001, one-way ANOVA. (**B**) In parallel experiments, binding of 25 μg/ml mCRP, generated from pCRP by urea/chelation treatment, was measured in DPBS without Ca^2+^ using the same polyclonal anti-CRP as in (A). Data are mean absorbance values ± SD from three experiments. ***p < 0.001, one-way ANOVA. (**C**) To measure CFHR5 binding to surface-bound CRP, 5 μg/ml CRP was immobilized in microplate wells, which results in the generation of mCRP. Equimolar amounts (300 nM) of CFHR5 and FH were added and their binding was detected using FH Ab. Data are mean absorbance values ± SD from four experiments. ***p < 0.001, one-way ANOVA. (**D**) The binding of 50 μg/ml (∼300 nM) FH to CRP, immobilized in microplate wells, was measured by ELISA in the presence of increasing concentrations of CFHR5 (up to 20 μg/ml, corresponding to ∼300 nM CFHR5) using an FH-specific mAb for detection. HSA was used as control protein. The normalized values are means ± SD derived from three independent experiments. The binding of FH was significantly different in the presence of 20 μg/ml CFHR5 than in the presence of 20 μg/ml HSA (*p* < 0.0001, unpaired *t* test). (**E**) The cofactor activity of FH bound to CRP for the cleavage of C3b in the presence and absence of CFHR5 was measured by Western blot. Microplate wells were coated with 10 μg/ml CRP and incubated with 50 μg/ml FH with or without 20 μg/ml CFHR5, as indicated below the blot, and then C3b and FI were added to each well. The blot was developed as described for Fig. 5 and is representative of three independent experiments. The molecular mass marker is indicated on the *left*, and the C3b chains and the C3b α′-chain cleavage fragments are indicated on the *right*.

CRP shares a pentraxin domain homologous to that of PTX3. CFHR5 was shown to interact with CRP, but the CRP form that binds to CFHR5 and the functional consequence of this interaction were not investigated (4). We therefore set out to characterize the CFHR5–CRP interaction in more detail. CFHR5 did not bind the native pCRP in ELISA under our experimental conditions (Fig. 6A), but it readily interacted with the denatured mCRP (Fig. 6B). The control proteins used in these experiments, CFHR4A and FH, bound pCRP and mCRP, respectively, as expected (16). CFHR5 also bound to mCRP in the reverse setting, when mCRP was generated by immobilization of pCRP on the ELISA plates (Fig. 6C).

Similar to PTX3, the binding of FH to mCRP was dose-dependently inhibited by CFHR5 (Fig. 6D). When bound to mCRP, FH displayed cofactor activity for the FI-mediated cleavage and inactivation of C3b (*lane 2*), which was inhibited by CFHR5 (*lane 3*) (Fig. 6E). CFHR5 itself showed no cofactor activity under these conditions (Fig. 6E, *lane 4*).

### CFHR5 binds to ECM, competes with FH, and recruits PTX3

**FIGURE 7.**
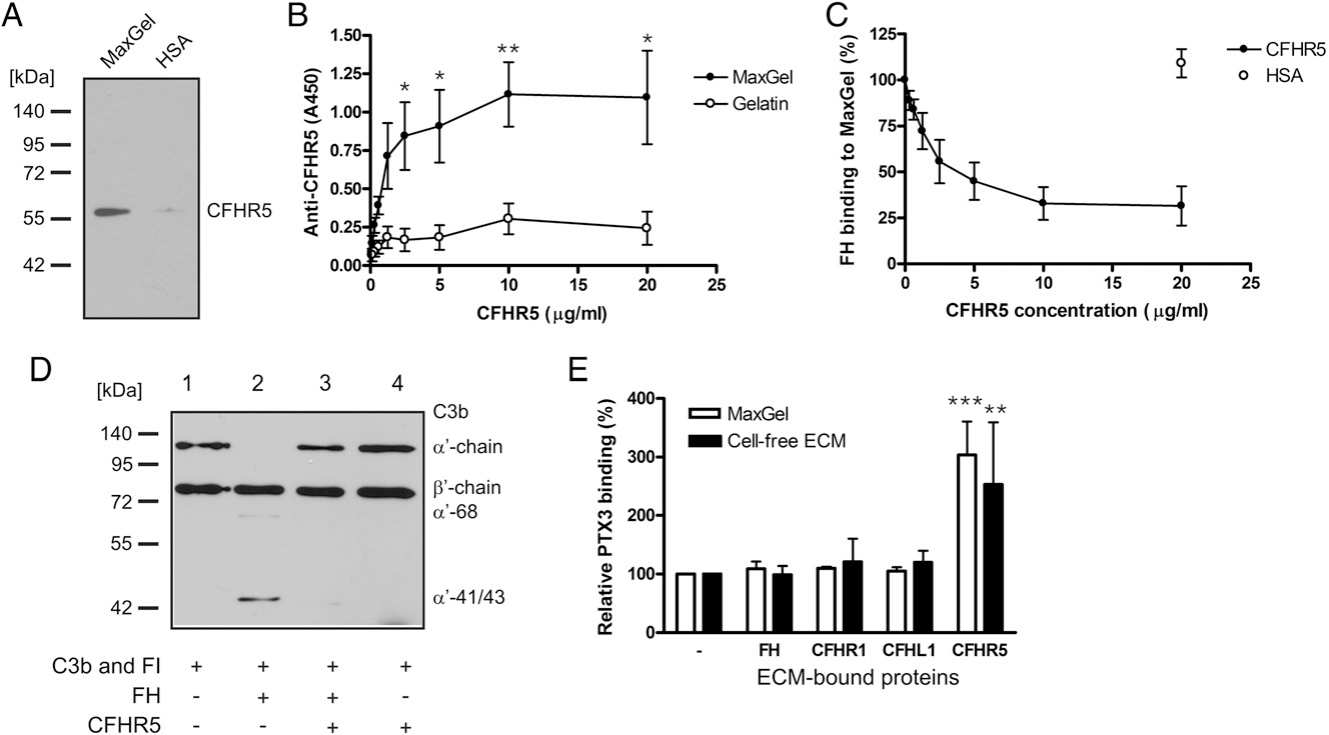
Interaction of CFHR5 with the extracellular matrix. (**A**) Microplate wells were coated with MaxGel or HSA and incubated with normal human serum. After washing, bound proteins were removed by adding SDS sample buffer and subjected to 10% SDS-PAGE and Western blotting using polyclonal anti-CFHR5. The blot is representative of three experiments. (**B**) Dose-dependent binding of recombinant CFHR5, added in the indicated concentrations, to MaxGel and gelatin (both immobilized at 20 μg/ml) was measured by ELISA using polyclonal Ab to human CFHR5. The data are means ± SD of absorbance values derived from four independent experiments. *p < 0.05, **p < 0.01, two-way ANOVA. (**C**) The binding of 50 μg/ml (∼300 nM) FH to MaxGel was measured by ELISA in the presence of increasing concentrations of CFHR5 (up to 20 μg/ml, corresponding to ∼300 nM CFHR5) using an FH-specific mAb for detection. HSA was used as control protein. The normalized values are means ± SD derived from three independent experiments. The binding of FH was significantly different in the presence of 20 μg/ml CFHR5 than in the presence of 20 μg/ml HSA (*p* = 0.0027, unpaired *t* test). (**D**) The cofactor activity of FH bound to MaxGel for the cleavage of C3b in the presence and absence of CFHR5 was measured by Western blot. Microplate wells were coated with MaxGel (diluted 1:30) and incubated with 50 μg/ml FH with or without 20 μg/ml CFHR5, as indicated below the blot, and then C3b and FI were added to each well. The blot was developed as described for Fig. 5 and is representative of three independent experiments. The molecular mass marker is indicated on the *left*, and the C3b chains and the C3b α′-chain cleavage fragments are indicated on the *right*. (**E**) Binding of 5 μg/ml PTX3 to MaxGel (open bars) and HUVEC-derived ECM (filled bars) preincubated with the indicated FH family proteins at 20 μg/ml (recombinant CFHR1 and CFHR5) or 100 μg/ml (recombinant FHL-1 and purified FH) concentration was measured by ELISA as described for Fig. 2. The normalized values are means ± SD derived from four independent experiments. **p < 0.01, ***p < 0.001, one-way ANOVA.

Previously, we showed that FH, FHL-1, and CFHR1 bind to ECM (27). Therefore, we analyzed the interaction of CFHR5 with the model ECM MaxGel. The binding of native CFHR5 to MaxGel from serum could be detected by Western blot (Fig. 7A). Recombinant CFHR5 exhibited a strong, dose-dependent binding to MaxGel (Fig. 7B). Similarly to the case of pentraxins (Figs. 5, 6), CFHR5 inhibited the surface-associated cofactor activity of FH by competing with its binding to MaxGel (Figs. 7C, 7D). Additionally, CFHR5, but not FH, FHL-1, and CFHR1, strongly enhanced binding of PTX3 to both MaxGel and HUVEC-derived ECM (Fig. 7E). PTX3 had only a minor effect on CFHR5 binding to ECM (not shown).

### CFHR5 competes with FH in serum

**FIGURE 8.**
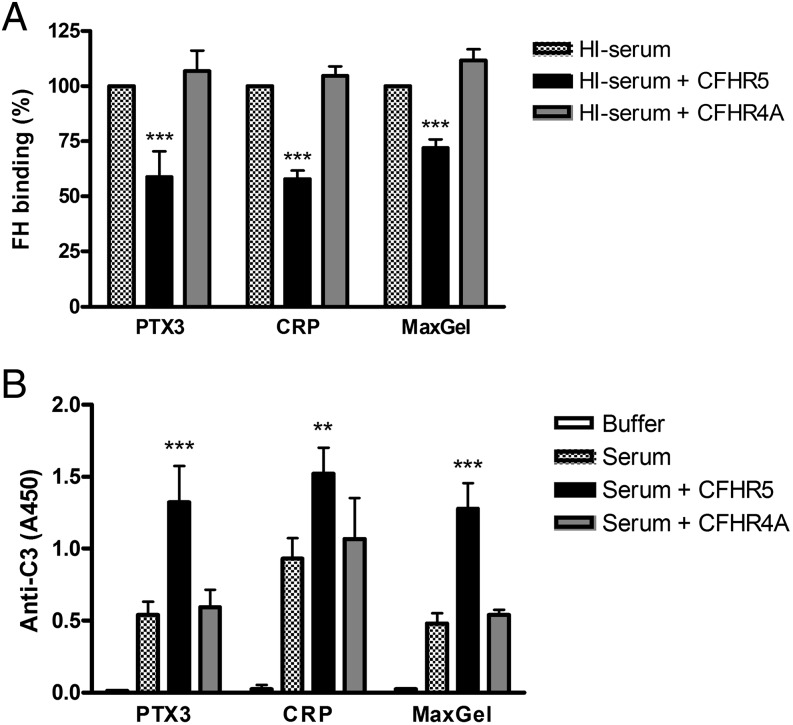
CFHR5 competes with FH in serum for binding to PTX3, CRP, and MaxGel and causes enhanced C3 fragment deposition. (**A**) Wells were coated with 10 μg/ml PTX3, 10 μg/ml CRP, and MaxGel diluted 1:30 in DPBS. After blocking, the wells were incubated for 30 min at 37°C with 25% heat-inactivated human serum with or without 0.5 μM CFHR5 and CFHR4A. FH binding was detected with mAb A254 and the corresponding secondary Ab. The normalized values are means ± SD derived from three independent experiments. ***p < 0.001, one-way ANOVA. (**B**) Nunc microplate wells were coated with 10 μg/ml PTX3, 10 μg/ml CRP, and MaxGel diluted 1:30 in DPBS. After blocking, 12.5% normal human serum (for PTX3 and CRP) and 25% normal human serum (for ECM) were added for 30 min at 37°C, with or without 20 μg/ml CFHR5 or CFHR4A. Complement activation was detected by measuring C3 fragment deposition using HRP-conjugated goat anti-human C3. Data represent mean absorbance values ± SD from three independent experiments. **p < 0.01, ***p < 0.001, one-way ANOVA.

To confirm that the observed competition between CFHR5 and FH occurs also in serum, wells were coated with PTX3, CRP, and MaxGel and incubated with heat-inactivated serum, which was used to exclude FH binding through deposited C3 fragments. CFHR5 when added to heat-inactivated serum significantly reduced the amount of bound FH on PTX3, CRP, and MaxGel (Fig. 8A). As a negative control, recombinant CFHR4A was used, which does not bind well to PTX3 (Fig. 2A), immobilized CRP (Fig. 6) (16), and MaxGel (27). In line with this, CFHR4A did not inhibit FH binding to any of these ligands (Fig. 8A).

Additionally, C3 fragment deposition on PTX3, CRP, and MaxGel was measured in complement active normal serum when CFHR5 was added to increase its concentration. In all cases, addition of CFHR5 but not CFHR4A caused increased C3 deposition, indicating competitive inhibition of FH activity by CFHR5 on these ligands (Fig. 8B).

### The C3bBb alternative pathway C3 convertase assembles on CFHR5

**FIGURE 9.**
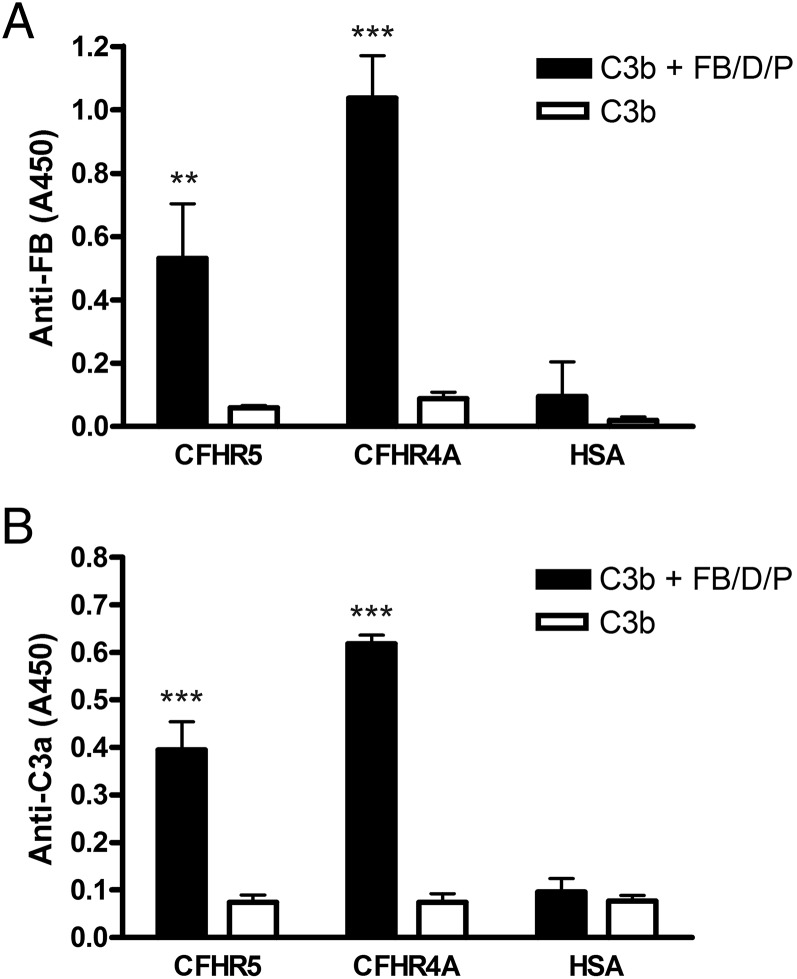
Assembly of the alternative pathway C3 convertase on CFHR5. (**A**) Assembly of the C3bBb convertase on CFHR5. Recombinant CFHR4A, CFHR5, and, as negative control, HSA were immobilized in microplate wells, followed by incubation with 10 μg/ml C3b. The alternative pathway C3 convertase was built up by adding purified FB, factor D, and FP for 30 min at 37°C. The convertase was detected with polyclonal anti-FB Ab. The data are mean absorbance values ± SD derived from five independent experiments. **p < 0.01, ***p < 0.001, one-way ANOVA. (**B**) Activity of the CFHR5-bound convertase was measured by adding 10 μg/ml C3 to the wells for 1 h at 37°C. C3a generation was measured by Quidel’s C3a ELISA kit. Data are mean absorbance values ± SD from three experiments. ***p < 0.001, one-way ANOVA.

We have previously shown that CFHR4 can activate complement by binding C3b and allowing formation of an active C3bBb alternative pathway C3 convertase (34). We analyzed whether such a C3 convertase can also be formed on CFHR5. To this end, immobilized CFHR5 was incubated with C3b, followed by the addition of purified FB, factor D, and FP. Convertase formation was detected by measuring the Bb fragment. Similar to CFHR4A, CFHR5 supported C3bBb formation, but the amount of C3 convertase was less on CFHR5 compared with CFHR4A (Fig. 9A). The CFHR5-bound convertase was functional as shown by the generation of C3a after the addition of purified C3 to the convertase (Fig. 9B). We detected no binding of purified FB alone to CFHR5. We observed weak binding of purified FP to CFHR5, which could be an artifact due to the known in vitro formation of nonphysiological FP oligomers (Supplemental Fig. 1).

**FIGURE 10.**
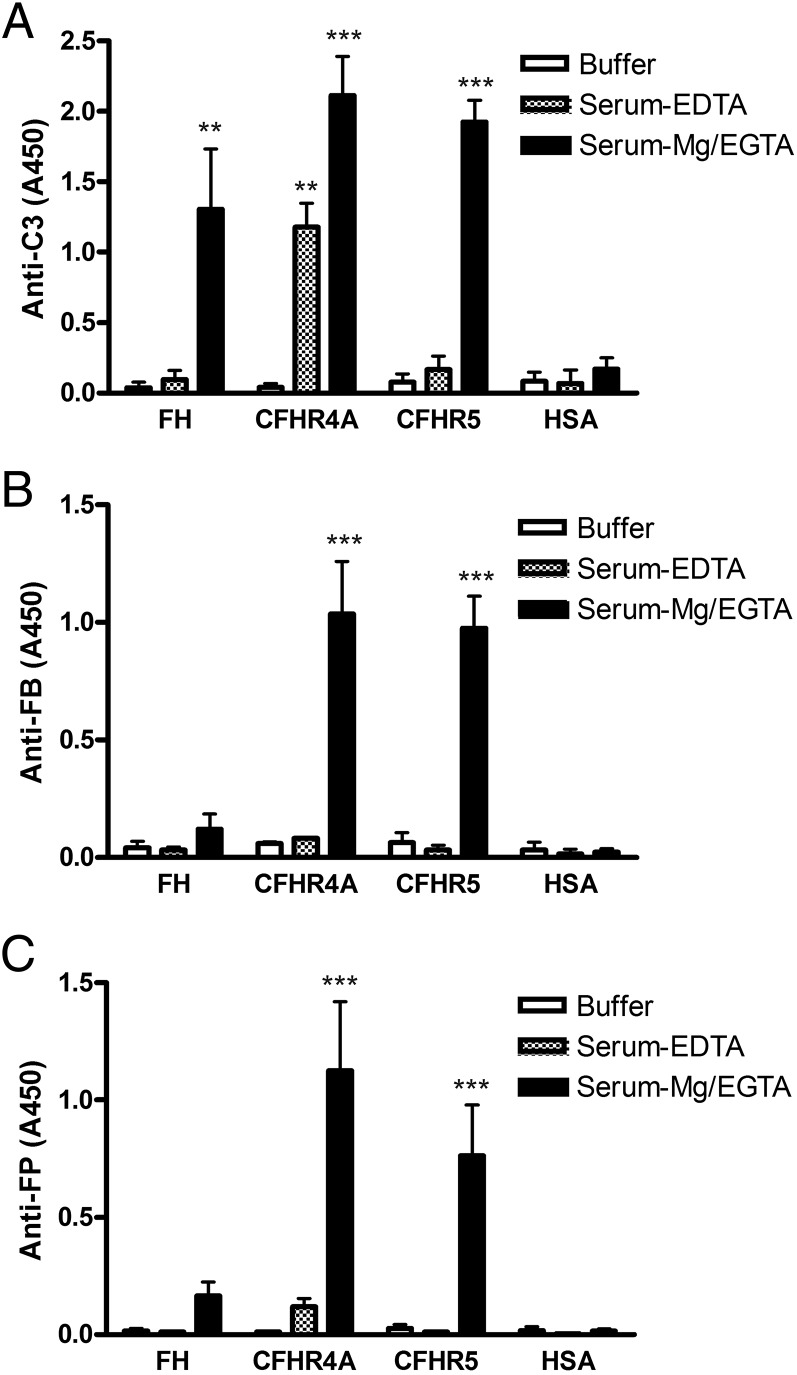
Complement activation by CFHR5. CFHR5 was immobilized on microplate wells and incubated with 10% normal human serum in 5 mM Mg^2+^-EGTA buffer to allow only alternative pathway activation or with 10% serum containing 5 mM EDTA to inhibit complement activation. Deposition of (**A**) C3b, (**B**) FB, and (**C**) properdin (FP) was detected using the corresponding Abs. Immobilized CFHR4A was used as positive control and FH and HSA were used as negative controls. The data are means ± SD derived from three independent experiments. **p < 0.01, ***p < 0.001, one-way ANOVA.

Incubation of CFHR5 with 10% normal human serum supplemented with 5 mM Mg^2+^-EGTA confirmed the generation of the alternative pathway C3 convertase as measured by deposition of C3, FB, and FP (Fig. 10). In serum supplemented with 5 mM EDTA, no complement activation and convertase formation was detected, as expected. The residual C3 signal on CFHR4A is due to direct binding of C3 fragments (Fig. 10A).

## Discussion

The function of the FH-related proteins is a controversial issue. Most studies reported lack of FH-like complement–inhibiting activity for the CFHR proteins at physiological concentrations (11, 12, 34, 36), as expected from the domain composition of these proteins. However, weak cofactor activity for CFHR3, CFHR4, and CFHR5 (4, 37) as well as strong cofactor activity for CFHR3 were reported (38). Complement regulation by other mechanisms, such as inhibition of the terminal complement pathway, were reported for CFHR1 and CFHR2 (39, 40), but others could not detect this activity (11, 41). Additionally, enhancement of complement activation by CFHR4 via binding of C3b and CRP was described (32, 34).

Despite the suggested complement-inhibiting function for the CFHRs, microbes that bound CFHR1, CFHR2, and CFHR5 were not protected by these proteins from complement attack (42, 43). Increased expression of CFHRs under infectious conditions (44) and the described competition of CFHR1 with FH for certain bacterial ligands (45, 46) and for the major opsonin C3b (11) indicate that CFHRs may interfere with the regulatory activity of FH. Cumulatively, these data suggest that instead of inhibition of complement activation, the CFHRs rather cause enhanced activation. This could be advantageous to the human host during infections, as it may help the opsonophagocytic removal of microbes, but also that of cellular debris under noninfectious conditions. Depending on the local concentration, oligomeric state, polymorphic and mutant variants, and binding strength of FH, CFHRs, and their ligands, the delicate balance between complement activation/deregulation and inhibition could be shifted in favor of activation, resulting in pathologic complement activation.

Although currently there is no convincing evidence for physiologically relevant complement-inhibiting roles of the CFHR proteins, their association with complement-mediated diseases is supported by genetic studies (47). Importantly, three recent reports showed that CFHR1, CFHR2, and CFHR5 deregulate complement by competing with FH for binding to C3b, and thus they may rather enhance complement activation in human renal diseases (11–13). Our present study supports a function of CFHR5 as competitive inhibitor of FH and as enhancer of complement activation. We further investigated the role of CFHR5 in complement activation and in modulating the activity of FH, which could be particularly relevant during acute phase response (with increase in pentraxin concentrations) and endothelial injury.

We identified novel CFHR5 ligands, such as PTX3, modified CRP, and ECM, which are likely relevant in disease-associated roles of CFHR5. CFHR5 was implicated in particular in various kidney pathologies based on genetic and protein-level studies (6–8, 10, 11, 48). Our data suggest that not only binding of C3b or C3b fragments (such as iC3b) by CFHR5 could be important in the context of diseases, but likely also interactions with molecules, such as pentraxins that are upregulated during inflammation and renal endothelial damage, and with host surfaces such as the ECM. This is supported by recent proteomics studies, which identified both CFHR5 and PTX3 as glomerular ECM-associated proteins (49, 50).

PTX3 is produced locally at the site of inflammation. Elevated PTX3 levels were described in various infectious and inflammatory diseases, including chronic kidney disease (23, 51, 52). Both protective and detrimental effects of PTX3 have been described, such as in postischemic renal injury (23, 53, 54). Systemic PTX3 concentration of up to ∼1.5 μg/ml was reported, indicating that locally at inflammatory sites it can reach much higher concentrations, similar to those used in this study, suggesting that the studied PTX3–CFHR5 interaction has physiological relevance.

A previous study described cofactor activity for CFHR5, but rather high concentrations of the protein were required for this function (4). In our present study, we found no cofactor activity of CFHR5 at the studied physiologically relevant concentrations (Figs. 5–7). However, physiological amounts of CFHR5 significantly competed with FH in binding to the pentraxins PTX3 and CRP, as well as to ECM. Importantly, this competition could be detected in serum and caused enhanced complement activation (Fig. 8). This represents an indirect means by which CFHR5 deregulates complement, namely, not via direct competition with FH for C3b, but by interfering with FH binding to its other physiological ligands. These data suggest that depending on the FH-binding surfaces and ligands, as well as on local FH and CFHR5 concentrations, CFHR5 can fine-tune local complement regulation by competing with FH.

The binding of PTX3 to CFHR5 is the strongest among the tested FH family proteins; this explains the strong inhibitory effect of CFHR5 on FH binding to PTX3 (Figs. 1, 2, 5). The PTX3–CFHR5 interaction is strongly reduced in Ca^2+^-free buffer (Fig. 2), similar to the PTX3 binding to FH, FHL-1, and CFHR1, as reported previously (26, 27).

Our data show that at reduced pH, which might occur at sites of inflammation, the binding of PTX3 to CFHR5 is reduced. We have previously shown that at lower pH the binding of PTX3 to C1q and CFHR1 is unchanged but the binding of PTX3 to the complement inhibitors FH, FHL-1, and C4b-binding protein is increased (27). Collectively, these data suggest that under such conditions complement inhibition is preferred, because the binding of the complement inhibitors increases and that of the deregulator CFHR5 decreases.

The PTX3 binding site in CFHR5 appears not to be homologous to the two identified PTX3 binding sites in FH, that is, FH domains CCP7 and CCP19–20. CCP1–2 of CFHR5 are the dimerization domains and very different structurally and functionally from CCP6–7 of FH. CCP2 of CFHR5 is very similar to CCP2 of CFHR1 (∼85% amino acid sequence identity); however, we showed previously that PTX3 binds to CFHR1 via the C terminus of CFHR1 (27). We tested the C-terminal domains of all of the five human CFHR proteins, and only CCP4–5 of CFHR1 bound PTX3 under our experimental conditions. The CCP8–9 domains of CFHR5 did not bind PTX3 (Fig. 3). Additionally, these domains were shown to bind C3b, and C3b did not influence PTX3 binding by CFHR5 in our experiments (not shown). Moreover, the binding site for the related pentraxin CRP was localized to CCPs 3–7 of CFHR5 (4). Thus, it is likely, but needs to be tested in the future, that the PTX3-binding site also resides within these middle domains of CFHR5. Additionally, the tested disease-associated mutant CFHR5 protein, in which the two N-terminal domains are duplicated, showed reduced PTX3-binding capacity in comparison with the wild-type protein (Fig. 3). This could be caused by the restricted availability of PTX3-binding sites in the mutant, which has an increased tendency to form oligomers (11).

The known PTX3 ligand C1q could partially inhibit PTX3 binding to CFHR5, but not to FH, indicating partially overlapping binding sites or conformational changes of PTX3 when bound to C1q. However, CFHR5-bound PTX3 could still strongly bind C1q (Fig. 4). We observed C1q binding to CFHR5 alone, which was strongly and significantly enhanced by PTX3.

Interaction of CFHR5 with CRP was first described by McRae et al. (4) but the type of CRP that binds CFHR5 was not studied. FH binds preferentially to the modified, monomeric form of CRP via domains CCP7, CCPs 8–11, and CCPs 19–20 (15–17), and at acute phase CRP concentrations it was reported to interact also with the native pentameric form of CRP (18). In contrast, CFHR4 binds primarily to the native pCRP (16, 32). In our assays, CFHR5 bound strongly to mCRP, whereas the binding to pCRP was minor (Fig. 6).

CFHR5 also strongly and dose-dependently bound to MaxGel, in contrast to the relatively weak binding of FH (27). Accordingly, CFHR5 efficiently inhibited the binding and cofactor activity of FH on this ECM (Fig. 7). CFHR5, alone among the investigated FH family proteins, recruited PTX3 to the ECM. These results are in line with recent proteomics data demonstrating the association of both CFHR5 and PTX3 with glomerular EC-derived ECM (49, 50), and they suggest that CFHR5 enhances local complement activation via these interactions.

We also studied the direct role of CFHR5 in complement activation. In our recent study on CFHR4, we showed that C3b binding to CFHR4 can result in the assembly of the alternative pathway C3 convertase and activation of C3 (34). In our present study, we found that CFHR5 can similarly promote C3 convertase formation and alternative pathway activation, albeit the effect was weaker compared with CFHR4 (Figs. 9, 10). This activity stands in contrast to the complement alternative pathway inhibitory role of FH.

During kidney endothelial injury, host ligands that are newly exposed (ECM) or the concentration of which is increased (pentraxins) may result in the increased binding of CFHR5 to the site of injury. The oligomeric state of CFHR5 and CRP can further fine-tune these interactions, but this needs to be characterized in more detail.

In summary, we show that in addition to competing for C3b binding with FH as reported recently, CFHR5 may promote complement activation and complement-mediated damage/inflammation through three mechanisms: 1) by preventing the binding of the complement inhibitor FH to PTX3, CRP, and ECM; 2) by allowing increased binding of the complement activation initiator molecule C1q; and 3) by supporting the assembly of the alternative pathway C3 convertase C3bBb. These functions may explain in part the disease-associated role of CFHR5.

## Supplementary Material

Data Supplement
