# Supplementary material for: Factor H–Related Protein 5 Interacts with Pentraxin 3 and the Extracellular Matrix and Modulates Complement Activation
Source: J Immunol. 2015 Apr 8;194(10):4963–73. doi: 10.4049/jimmunol.1403121 (PMC4416742; doi:10.4049/jimmunol.1403121)
Supplement: Data Supplement [file JI_1403121.zip › JI_1403121_Supplemental_Figure_1.pdf]

Csincsi et al.

Supplemental Figure 1

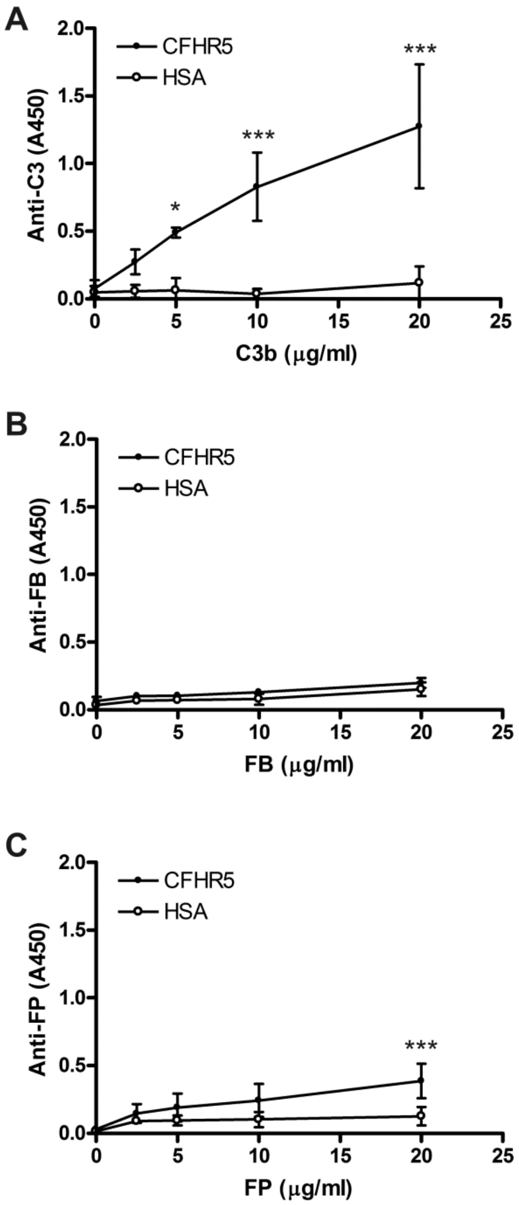

Figure S1. Binding of purified C3b, factor B (FB) and properdin (FP) to CFHR5.

Dose-dependent binding of C3b (A), FB (B) and FP (C), added in the indicated concentrations, to CFHR5 and HSA, both immobilized at 5 µg/ml, was measured by ELISA using the corresponding antibodies. Data are means ± SD from at least three independent experiments. *, $p < 0.05$ and ***, $p < 0.001$ (two-way ANOVA).
